# Circulating Biomarkers of Endothelial Dysfunction and Inflammation in Predicting Clinical Outcomes in Diabetic Patients with Critical Limb Ischemia

**DOI:** 10.3390/ijms231810641

**Published:** 2022-09-13

**Authors:** Francesco Vieceli Dalla Sega, Paolo Cimaglia, Marco Manfrini, Francesca Fortini, Luisa Marracino, Davide Bernucci, Graziella Pompei, Antonella Scala, Michele Trichilo, Beatrice De Carolis, Luca Dalla Paola, Roberto Ferrari, Paola Rizzo, Gianluca Campo

**Affiliations:** 1 Maria Cecilia Hospital, GVM Care and Research, 48033 Cotignola, Italy; 2 Department of Translational Medicine, Laboratory for Technologies of Advanced Therapies (LTTA), University of Ferrara, 44121 Ferrara, Italy; 3 Cardiology Unit, Ospedale M. Bufalini, 47521 Cesena, Italy; 4 Cardiology Unit, Azienda Ospedaliero-Universitaria di Ferrara, University of Ferrara, 44124 Ferrara, Italy

**Keywords:** endothelial dysfunction, diabetes, critical limb ischemia, peripheral artery disease, wound healing, diabetic foot ulcer

## Abstract

Critical limb ischemia (CLI) is a severe manifestation of peripheral artery disease characterized by ischemic pain, which is frequently associated with diabetes and non-healing lesions to inferior limbs. The clinical management of diabetic patients with CLI typically includes percutaneous transluminal angioplasty (PTA) to restore limb circulation and surgical treatment of diabetic foot ulcers (DFU). However, even after successful treatment, CLI patients are prone to post-procedure complications, which may lead to unplanned revascularization or foot surgery. Unfortunately, the factors predicting adverse events in treated CLI patients are only partially known. This study aimed to identify potential biomarkers that predict the disease course in diabetic patients with CLI. For this purpose, we measured the circulating levels of a panel of 23 molecules related to inflammation, endothelial dysfunction, platelet activation, and thrombophilia in 92 patients with CLI and DFU requiring PTA and foot surgery. We investigated whether these putative biomarkers were associated with the following clinical endpoints: (1) healing of the treated DFUs; (2) need for new revascularization of the limb; (3) appearance of new lesions or relapses after successful healing. We found that sICAM-1 and endothelin-1 are inversely associated with DFU healing and that PAI-1 and endothelin-1 are associated with the need for new revascularization. Moreover, we found that the levels of thrombomodulin and sCD40L are associated with new lesions or recurrence, and we show that the levels of these biomarkers could be used in a decision tree to assign patients to clusters with different risks of developing new lesions or recurrences.

## 1. Introduction

Peripheral artery disease (PAD) is a manifestation of atherosclerosis in which the narrowing of peripheral arteries causes a reduced blood flow in the limbs, producing symptoms such as weakness, claudication, numbness, and pain [1]. Diabetes is a crucial risk factor for PAD and is associated with disease severity. In the most severe cases, PAD can progress to critical limb ischemia (CLI), characterized by chronic ischemic rest pain and diabetic foot ulcers (DFUs). If not treated, these lesions can cause tissue loss, requiring lower extremity amputations in the worst scenario [2]. The management of patients with CLI and DFU requires a multidisciplinary approach, including percutaneous transluminal angioplasty (PTA), to restore limb circulation, and surgical treatments of wounds, which are typically performed at specialized diabetic foot units [3]. However, the post-procedure course is often complicated: even when treated with the best practices, the wounds can take months to heal, and limb circulation can deteriorate, requiring revascularization of the same vessel or different sites [4]. Moreover, even after successful healing, recurrences of previously treated wounds or the emergence of new lesions are frequent [5]. Based on wound features and ischemia severity, CLI patients with DFU are considered at high risk of adverse events such as amputation or incomplete healing [6]. Unfortunately, reliable methods for a more accurate stratification of individual risk in this population are lacking [7]. Therefore, global vascular guidelines on the management of CLI have included the identification of biomarkers predicting clinical events among the research priorities [3]. 

Several processes participate in the pathophysiology of CLI in diabetic patients, including endothelial dysfunction, altered immune response, increased platelet activity, and coagulation abnormalities [8]. Crucially, these factors are also associated with defective wound healing in diabetes and, hence, linked to complications such as DFU [9,10,11].

This investigation aimed to identify the circulating factors associated with poor prognosis in patients with CLI and diabetes enrolled in the “Prospective Evaluation of Laboratory, Cellular and Molecular Determinants of Clinical Success of the Treatment in Diabetic Patients With Critical Limb Ischemia” (ECAD-CLI) study.

## 2. Results

### 2.1. Study Cohort and Clinical Outcomes 

From August 2018 to October 2019, 92 diabetic patients were admitted to the hospital with CLI and DFU requiring PTA and foot surgery, fulfilling the criteria for inclusion in the study. The average age of the population was 72.5 years; 77% of the patients were males, and 93% had type 2 diabetes. All the patients were at moderate (20%) or high risk (80%) of amputation according to the Society for Vascular Surgery Wound, Ischemia, and foot Infection (WIfI) classification [6]. The characteristics of the population including co-morbidities, medical history, and the type of interventions are reported in Table 1 and Table 2. At the three-month follow-up, 52% of the patients showed optimal wound healing. However, during the 12 months following limb revascularization, 25.8% of the patients required a new PTA and 42.7% developed a new lesion or had recurrences at the same site after healing. 

To investigate potential factors associated with each of the three endpoints, we performed Cox regression for each variable including co-morbidities, standard laboratory parameters and a panel of 23 potential biomarkers selected among molecules related to inflammation, endothelial dysfunction [12], platelet activation, and thrombophilia (Table 1).

We found that the sICAM-1 (HR: 0.764; CI: 0.604–0.965; *p* = 0.0239) and endothelin-1 (HR: 0.208; CI: 0.048–0.901; *p* = 0.0358) levels were inversely associated with healing within three months. By contrast, we observed a positive association between healing within three months and the levels of albumin (HR: 1.709; CI: 1.181–2.473; *p* = 0.0045), lymphocytes (HR: 1.396; CI: 1.089–1.789; *p* = 0.0085), and monocytes (HR: 1.152; CI: 1.023–1.297; *p* = 0.0196). 

Regarding the need for a new revascularization, we found that the levels of PAI-1 (HR: 1.893; CI: 1.127–3.179; *p* = 0.0159) and endothelin-1 (HR: 7.457; CI: 1.093–50.859, *p* = 0.0402) were associated with increased risk. Furthermore, we observed that the presence of atrial fibrillation was associated with this endpoint (HR: 2.376; CI: 1.028–5.495; *p* = 0.043). 

Finally, the occurrence of new lesions or recurrences were positively associated with the levels of IL-10 (HR: 42.136; CI: 3.799–467.359; *p* = 0.002), IL1RA (HR: 1.169; CI: 1.006–1.359; *p* = 0.042), and sCD40L (HR: 1.744; CI: 1.055–2.882; *p* =0.030). On the contrary, the levels of thrombomodulin were inversely associated with this event (HR 0.453; CI: 0.258–0.797; *p* = 0.006). Among other variables, we observed a positive association with the presence of atrial fibrillation (HR: 2.429; CI: 1.22–4.834; *p* = 0.012) and monocyte count (HR: 1.268; CI: 1.12–1.435; *p* = 0.001). The variables that were significantly associated with each of the endpoints are summarized in Table 3.

### 2.2. Clustering and Partition Tree

To identify a combination of the parameters that could effectively identify the CLI patients at a higher risk for adverse events, we first clustered patients using a semi-supervised approach, then we developed a partition tree to assign patients to the respective clusters. For each endpoint, the variables that we had previously identified with Cox regression (Table 3) were used for the clustering. The characteristics of the clusters for each of the three endpoints are summarized in Supplementary Tables S1–S3.

**Figure 1:**
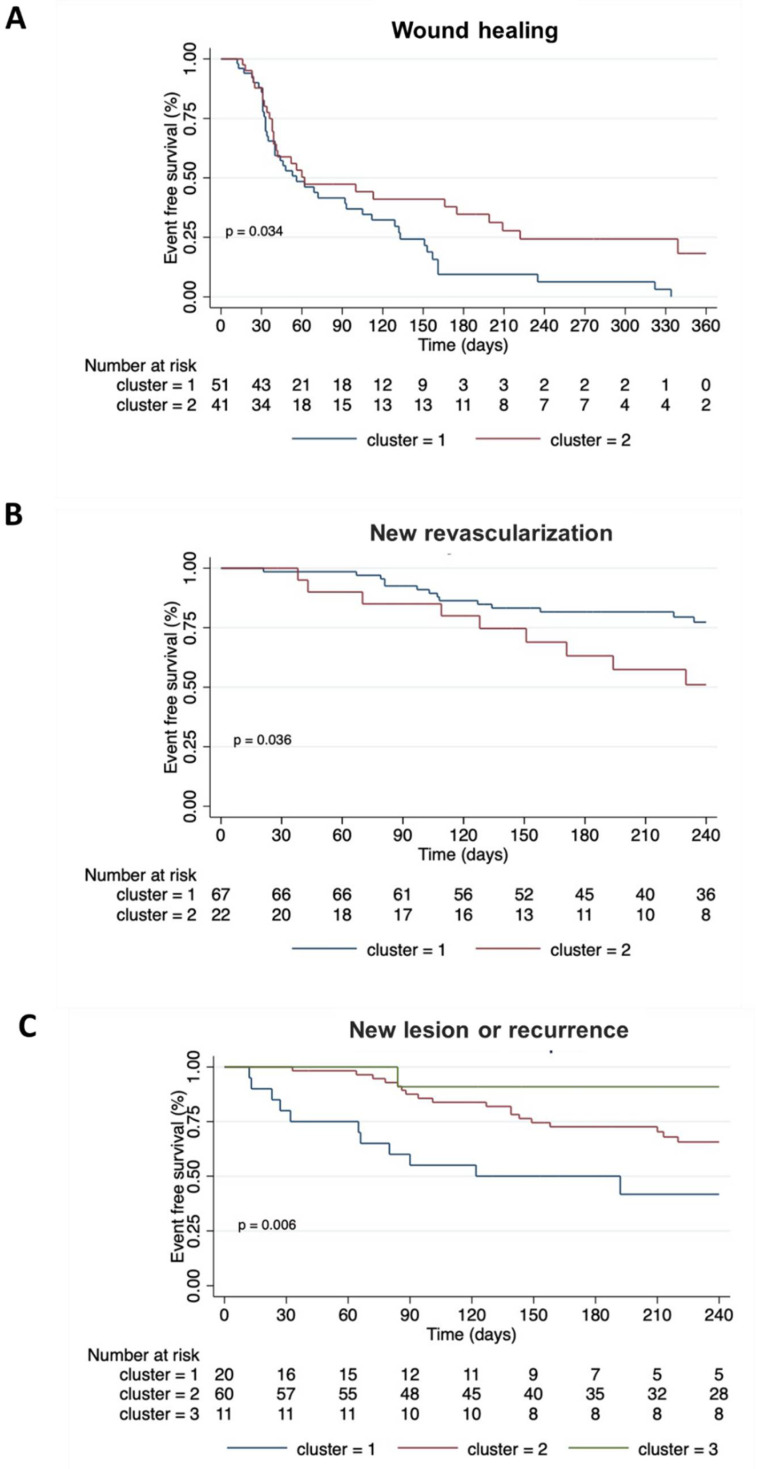
Event-free survival curves obtained by the Kaplan–Meier estimate showing significant differences between clusters toward: (**A**) DFU healing within three months from treatment; (**B**) new lower limb revascularization; (**C**) new lesions or recurrences. The log rank test was performed to compare survival functions. For each cluster, the number of event-free patients at any given time during follow-up is shown below the graphs.

For the first outcome (healing of DFU within three months), we obtained two clusters (n1 = 51, n2 = 41). Cluster 1 in comparison to cluster 2 was characterized by a higher frequency of atrial fibrillation patients and lower levels of IFN-γ and hemoglobin (Table S1). Cox regression showed that cluster 2 was associated with the healing outcome (HR = 0.587; 95% CI = 0.358–0.963; *p* = 0.035). As shown in Figure 1A, the survival curves confirmed that patients in cluster 2 had better healing over time (*p* = 0.034). For the second outcome (need for new revascularization), two clusters (n1 = 70, n2 = 22) were identified. For this endpoint, the presence of atrial fibrillation outclassed the other factors, and as a result, all the patients belonging to cluster 2 presented this condition (Table S2). Cox regression showed that cluster 2 had an increased risk of PTA outcome (HR = 2.376; 95% CI = 1.028–5.495; *p* = 0.043). The Kaplan–Meier curves in Figure 1B show that patients of cluster 2 had an increased risk of needing a new PTA within 12 months (*p* = 0.036). 

Three clusters were obtained for the third outcome (new lesion or recurrences) (n1 = 21, n2 = 60, n3 = 11). The levels of several biomarkers varied significantly between clusters, and the most pronounced differences regarded thrombomodulin and scDL40. Moreover, patients at the highest risk (cluster 1) had the highest levels of monocytes and glycated hemoglobin (Table S3). Of note, these clusters were associated with a new or recurrent lesion outcome as follows: compared to the reference cluster 1, cluster 2 showed an HR = 0.437 (95% CI = 0.218–0.877; *p* = 0.02) and cluster 3 showed an HR = 0.07 (95% CI 0.009–0.551; *p* = 0.012). Of note, also after adjustment for potential confounders including age and previous myocardial infarction, clusters 2 and 3 showed a decreased risk in comparison to cluster 1 (HR = 0.46; 95% CI = 0.23–0.95; *p* = 0.03 and HR = 0.07; 95% CI= 0.01–0.57; *p* = 0.01, respectively). Survival curves confirmed that event-free survival was different between the three clusters (*p* = 0.006), as shown in Figure 1C. 

**Figure 2:**
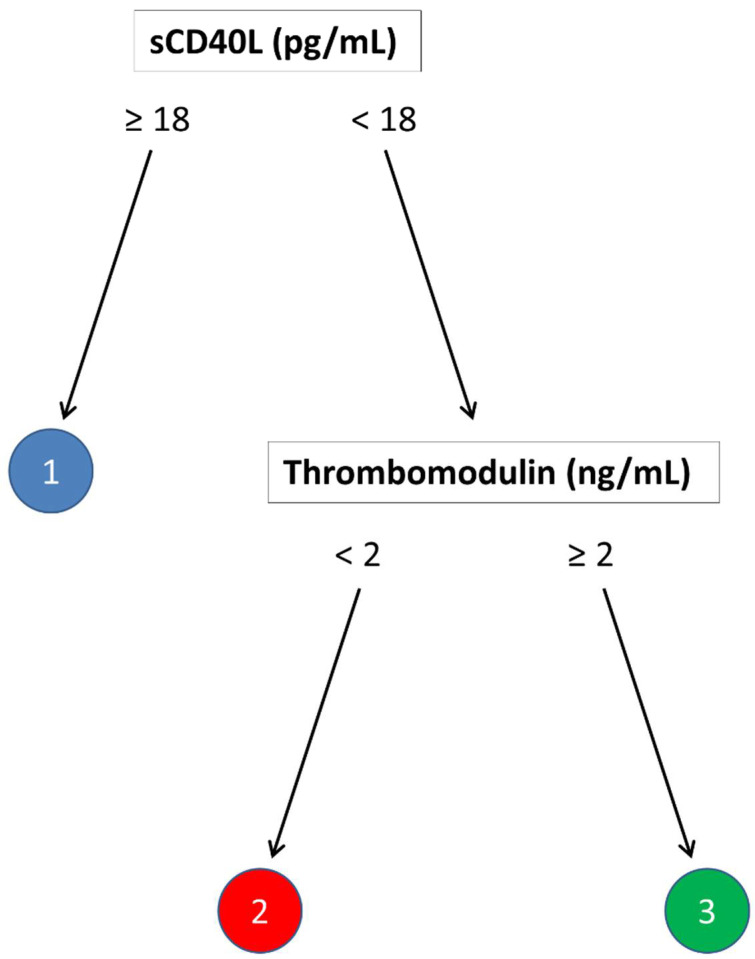
Decision tree obtained by cross-validation of the training set. The splits classify patients into three clusters with a different risk of new lesion or relapse outcome.

For each endpoint, we developed a partition tree to identify the variables that can differentiate between different clusters. We used a training set containing 65 cases, while the remaining 27 cases constituted the test set. We used the maximum accuracy value to select the optimal model. For the first and second endpoints, the tree that emerged had only one split, possibly due to the limited number of variables identified with Cox regression. For the third endpoint, we developed a tree model with two splits based on sCD40L and thrombomodulin levels. As illustrated in Figure 2, sCD40L levels of less than 18 ng/mL determined cluster 1; those patients with more than 18 pg/mL could, in turn, be attributed to clusters 2 or 3 based on levels of thrombomodulin higher or lower than two ng/mL, respectively. When applied to the test set, the overall accuracy of this model was 0.812 (95% CI = 0.6192–0.937). More details of the partitioning tree and its performance on the test set are reported in the Supplementary.

## 3. Discussion

In this study, we investigated the possible usefulness of a panel of 23 selected biomarker proteins involved in inflammation, endothelial dysfunction, platelet activation, and thrombophilia, in addition to standard clinical laboratory and clinical parameters, in predicting the prognosis of diabetic patients with CLI undergoing limb vascularization and surgical treatment of DFU. 

We found that the sICAM-1 and endothelin-1 levels were inversely associated with the healing of the wounds within three months. Interestingly, both of these molecules are expressed by endothelium, and their release is associated with endothelial dysfunction [12]. Increased sICAM-1 is associated with inflammation and reflects endothelial activation or damage. Endothelin-1 acts on vascular smooth muscle cells, acting as a potent vasoconstrictor. In fact, high endothelin-1 levels are associated with the impairment of vascular tone regulation in diabetes [13]. These findings are in agreement with evidence showing that endothelial dysfunction can disrupt or delay the healing process in CLI patients [9]. Furthermore, in our cohort, the odds of healing of treated DFU were associated with the levels of albumin, leukocytes, and monocytes, in line with other studies showing an association between albumin and DFU healing [14] and with the well-known role of monocyte orchestrating this process [11].

Of note, PAI-1 and endothelin-1 levels were associated with the need for revascularization within 12 months from the previous treatment. PAI-1 is produced by endothelial cells, and its elevation is associated with endothelial dysfunction and is a risk factor for thrombosis and atherosclerosis [15], again a data point to a link between endothelial dysfunction and a poor prognosis in CLI patients. 

Furthermore, the levels of IL-10, IL1RA, and CD40L and the number of monocytes were linked with an increased risk of developing new lesions or recurrences after DFU healing. On the contrary, thrombomodulin levels were inversely associated with this endpoint. It is well known that in response to inflammation, monocytes are recruited to the wounds, where they differentiate into macrophages participating in the healing process [16]. IL-10 can be produced by monocytes, macrophages, or T-cells, and it is known for its immunomodulatory activity [17]. Interestingly, M2 macrophages are regarded as immunosuppressive cells and are the main source of IL-10 in the wound [18,19]. Recently, it has been elegantly demonstrated that M2 macrophages are predominant in DFUs, which do not heal [20]. 

Overall, our data align with existing literature suggesting that defective innate immune response, as indicated by high IL-10/M2 macrophages, may be involved in recurrences or new lesions. Elevated release of sCD40L has been observed in diabetic patients and PAD [21,22], and it is associated with the number of lower extremity diseased segments [23]. CD40L can be expressed by several immune cell types that regulate macrophage activity [24]. Interestingly, CD40L is able to promote macrophages’ expression of metalloproteinase-1 (MMP-1), a collagenase that is overexpressed in long-healing DFU [25]. 

Finally, we found that circulating thrombomodulin (TM) levels were inversely associated with the emergence of new lesions or recurrences. TM is expressed by the endothelium in response to an inflammatory stimulus. TM can be released from endothelial cells following a proteolytic cleavage. For this reason, increased levels of soluble TM are usually regarded as a marker of endothelial damage [12,26]. However, the physiological role of TM is suppressing excessive inflammation and coagulation, and it has been shown that increased circulating TM is associated with a reduced number of cardiovascular events [27,28]. Importantly, TM promotes wound healing regulating keratinocyte differentiation and angiogenesis [29,30]. In our cohort, TM levels were associated with a decreased risk of recurrences or new lesions, suggesting that TM may favor the healing processes in CLI patients.

The semi-supervised clustering using the variables associated with each of the clinical outcomes allowed us to obtain risk groups with different characteristics linked to the risk of the corresponding endpoint. In addition, for each endpoint, we developed a partition tree to identify the variables that can differentiate between different clusters. However, we were able to develop a decision tree with more than one split only for the endpoint of recurrences and new lesions. This is possibly due to a limited number of variables identified with Cox regression for the first two endpoints (DFU healing and the need for new revascularization). Of note, regarding the occurrence of new lesions or recurrences, we were able to develop a decision tree including two splits: sCD40L levels of more than 18 ng/mL determined cluster 1; patients with less than 18 ng/mL were attributed to either cluster 2 or 3 based on levels of TM higher or lower than 2 ng/mL, respectively. This tree displayed a good general performance in classifying patients, with an accuracy of about 80% for the outcome. No decision tree model was successfully inferred from the data toward the other two outcomes (DFU healing and need for new revascularization) because of the lack of their classification capabilities, suggesting that in our cohort, the selected biomarkers may not add relevant information to standard parameters.

Although this study was primarily focused on new biomarkers, we also observed some associations between clinical history and outcomes. Notably, atrial fibrillation was associated with the need for new revascularization, as emerged both by Cox regression and clustering analysis. This is in line with findings showing that concomitant PAD and atrial fibrillation are associated with the risk of cardiovascular adverse events [31]. In addition, we observed that patients belonging to the cluster at the highest risk of new lesions had the highest levels of glycated hemoglobin. This is consistent with the well-documented effect of hyperglycemia in wound healing [32]. 

This is a pilot study that has limitations. First, the size of the population, even if comparable with similar longitudinal studies, was relatively small. Second, the decision tree was developed and tested in the same population from a single center. Third, for only one endpoint we were able to develop a decision tree based on more than one variable, probably as a result of limited statistical power. Finally, although possible confounding variables were taken into account, it must be acknowledged that in a complex population such as CLI patients with foot lesions, there may be interactions between clinical and laboratory variables that were not captured in this study. For these reasons, this study has to be regarded as hypothesis-generating, and these findings need validation in a larger cohort.

In conclusion, in this work, we showed that biomarkers of inflammation and endothelial dysfunction are associated with the risk of delayed healing and post-procedure complications in diabetic patients with CLI. Furthermore, we provided a proof of concept of their potential practical utility in defining individual risk in these patients. Further studies on larger cohorts may confirm the usefulness of these molecules, individually or jointly, in the stratification of the risk of CLI patients.

## 4. Materials and Methods

### 4.1. Clinical Study

The ECAD-CLI (NCT03636867) is an investigator-driven, single-center, prospective, single-arm study enrolling patients admitted to the Diabetic Foot Unit of the Maria Cecilia Hospital (Cotignola, Italy) with a diagnosis of diabetes mellitus and critical limb ischemia (CLI) with DFU (consistent with the Rutherford classes 5 or 6). All patients required PTA and foot surgery. Exclusion criteria were acute limb-threatening ischemia, trauma, non-atherosclerotic disease (e.g., arteritis), embolic disease, known hypercoagulable state, or any acute cardiac disease (e.g., acute coronary syndrome, heart failure, unstable arrhythmias) or acute non-cardiac disease (e.g., severe sepsis, acute kidney disease, pneumonia) that may alter inflammatory markers. The study aimed to prospectively collect clinical, laboratory, angiographic, cellular, and molecular variables related to prognosis and outcome in patients with diabetes mellitus and CLI. Clinical and procedural data from all patients are registered in a dedicated electronic database. The first patient was included in August 2018. Patients were prospectively followed for 12 months. The protocol of the study follows the Declaration of Helsinki. All participants provided written informed consent. The present analysis considered the first 92 enrolled patients, and it focused on circulating levels of a panel of 23 molecules related to inflammation, endothelial dysfunction, platelet activation, and thrombophilia. Given the exploratory nature of this study, the sample size was not formally calculated.

### 4.2. Follow-Up and Endpoints 

Patients returned for surgical visits every 15 days until clinical stabilization of the surgical site. Afterward, they were followed up for 12 months, with visits every three months. During the visits, a physical examination was performed, and adverse events and compliance with medical therapy were assessed. For the present analysis, we considered three endpoints: (i) healing of a treated DFU after three months; (ii) need for a new limb revascularization; (iii) appearance of new lesions or relapses after successful healing of the DFU. The endpoints were defined as follows: (i) a treated DFU was considered healed when full epithelialization was achieved and the wound remained closed for at least four weeks; (ii) the need for new revascularization was defined as the necessity to execute an unplanned PTA on the lower limbs during the follow-up period; (iii) a new lesion was defined as the occurrence of a new wound at a site other than the treated DFU; (iv) a relapse was defined as a wound occurring at the same site of the treated DFU after a successful healing.

### 4.3. Collection of Specimens

At admission to the hospital, prior to PTA and foot surgery, blood samples were collected in Vacutainer serum separator tubes (BD Medical, Franklin Lakes, NJ, USA), and the serum was separated following standard procedures. Aliquots were stored at −80 °C prior to the analysis.

### 4.4. Biomarkers Assessment

The serum levels of soluble CD40 ligand (sCD40L), interferon (IFN)-α2, IFN-γ, IL- interleukin-1 receptor antagonist (1RA), IL-2, IL-4, IL-5, IL-6, IL-10, IL-13, IL-18, tumor necrosis factor alpha (TNF-α), Angiopoietin-2, Endoglin, Endothelin-1, soluble endothelial selectin (sE-Selectin), Thrombomodulin, soluble receptor for advanced glycation end-products (s-RAGE), soluble Intercellular Adhesion Molecule 1 (sICAM-1), P-Selectin, soluble vascular cell adhesion molecule 1 (sVCAM-1), and Plasminogen activator inhibitor-1 (PAI-1) were determined with the bead-based multiplex immunoassay Milliplex Map (EMD Millipore, Burlington, MA, USA). Samples were processed following the manufacturer’s instructions, and data were analyzed by the MAGPIX system provided with the xPONENT Software (Luminex, ThermoFisher Scientific, Waltham, MA, USA). The von Willebrand factor (vWF) was measured with a commercial ELISA kit (Thermofisher, Waltham, MA, USA) following the manufacturers’ instructions.

### 4.5. Statistics

The continuous variables are presented as the median and [interquartile range] and categorical variables as counts and proportions (%). For continuous variables, the differences were compared between groups using the one-way analysis of variance and the Kruskal–Wallis test for parametric and non-parametric data, respectively. Cox proportional hazards regression modelling was used to analyze the effect of all variables in a univariate model for each of the three endpoints. The results are reported as hazards ratios and associated 95% confidence intervals (CIs). The analysis was performed by M.M. with R version 3.5.1 (R Foundation for Statistical Computing, Vienna, Austria) and STATA.

### 4.6. Patients Clustering and Partition Tree

The Cox regression was employed to select the patient characteristics associated with each outcome. The sets of the identified variables were employed in the semi-supervised clustering of patients toward each outcome. The optimal number of clusters (k) was determined by calculating the Gower distance and then applying the silhouette method to the partitioning around medoids (PAM) algorithm. With the same distance metric and applying the PAM clustering, we partitioned cases into specific risk groups. To assess the relative risk of each cluster, the cluster variable was regressed toward each outcome. For partition tree development, the sample was partitioned into training and test sets by setting a ratio of 7: 3 for the cluster variable obtained with the method described above. A classification tree was trained by cross-validation (n = 10) using the set of variables selected by the Cox regression as described above for the semi-supervised procedure. The ability to predict the correct cluster class of each patient was evaluated on the test set. We used a confusion matrix to evaluate the following classifiers: accuracy, sensitivity, and specificity [33]. 

## Figures and Tables

**Table 1:** Characteristics of the population at the baseline. Continuous variables are expressed as the median and interquartile range. Categorical variables are expressed as the number and percentage of the population. Abbreviations: body max index (BMI); transient ischemic attack (TIA); percutaneous transluminal angioplasty (PTA); estimate of glomerular filtration rate (EGFR); high-density lipoprotein (HDL); low-density lipoprotein (LDL); glycated hemoglobin (Hba1c); c-reactive protein (CRP).

Age	72.50 [68.00–78.00]
Sex male	71 (77.2%)
BMI	26.68 [24.93–29.30]
Current smoker	9 (9.9%)
Former smoker	55 (60.4%)
Diabetes type II	85 (93.4%)
Insulin therapy	67 (76.1%)
Carotid artery disease	8 (8.7%)
Stroke or TIA	8 (8.8%)
Heart failure	8 (8.7%)
Coronary artery disease	42 (45.7%)
Previous myocardial_infarction	10 (10.9%)
Valvular prosthesis	4 (4.4%)
Atrial fibrillation	22 (23.9%)
**Biomarkers**	
sCD40L pg/mL	11.73 [7.32–17.91]
IFN-α2 pg/mL	8.00 [8.00–8.00]
IFN-γ pg/mL	1.28 [1.28–3.00]
IL-1Ra pg/mL	7.56 [3.82–12.47]
IL-2 pg/mL	0.64 [0.64–0.64]
IL-4 pg/mL	1.60 [0.64–3.86]
IL-5 pg/mL	3.54 [2.29–6.88]
IL-6 pg/mL	4.83 [2.15–9.08]
IL-10 pg/mL	2.56 [2.56–2.56]
IL-13 pg/mL	6.40 [6.40–10.12]
IL-18 pg/mL	34.01 [23.73–46.45]
TNF-α ng/mL	19.14 [12.00–30.75]
Angiopoietin-2 ng/mL	2.92 [1.92–4.51]
Endoglin ng/mL	1.71 [1.31–2.15]
Endothelin-1 pg/mL	2.70 [2.70–2.70]
E-Selectin ng/mL	34.19 [23.82–42.13]
Thrombomodulin ng/mL	1.13 [0.82–1.82]
sRAGE pg/mL	56.21 [31.90–97.12]
sICAM-1 ng/mL	201.71 [154.75–263.22]
P-Selectin ng/mL	102.77 [74.50–139.38]
sVCAM-1 ng/mL	1036.44 [870.09–1239.68]
PAI-1 ng/mL	189.48 [147.70–241.46]
vWF µg/mL	26.85 [17.90–40.02]
**Laboratory**	
White blood cells	9.05 [7.25–10.72]
Hemoglobin	12.10 [10.80–13.22]
Platelets	277.50 [216.75–333.00]
Neutrophils	6.40 [4.68–7.80]
Lymphocytes	1.80 [1.40–2.20]
Monocytes	0.70 [0.50–0.80]
EGFR (Cock)	58.69 [42.70–84.93]
Triglycerides	121.00 [89.75–161.50]
HDL	38.00 [33.00–46.00]
LDL	65.60 [51.50–87.80]
Hba1c	7.32 [6.34–8.12]
CRP	1.20 [0.25–3.20]
Albumin	3.62 [3.29–3.98]

**Table 2:** Characteristics of the lesions and interventions. Variables are expressed as the number of patients and percentage of the population. Wound and ischemia grades are reported according to the Society for Vascular Surgery Wound, Ischemia, and foot Infection (WIfI) classification [6]. As some patients required more than one type of surgery or revascularization, the sum of patients of the different types of treatments exceeds 100%. Abbreviations: above the knee (ATK); below the knee (BTK).

**Wound Classification and Treatment**	
**Wound severity**	
1	33 (36%)
2	58 (63%)
3	1 (>1%)
**Ischemia grade**	
2	7 (8%)
3	85 (92%)
**Foot infection**	
0	6 (7%)
1	54 (59%)
2	31 (34%)
3	1 (>1%)
**Risk of amputation (WIfi)**	
Moderate	18 (20%)
High	74 (80%)
**Lesion site**	
Digits	58 (63%)
Forefoot	14 (15%)
Midfoot	6 (7%)
Hindfoot	9 (10%)
Ankle	3 (3%)
Leg	2 (2%)
**DFU treatment**	
Amputation	34 (37%)
Sequestrectomy	36 (39%)
Debridement or ulcerectomy	57 (62%)
Dermal regeneration template	18 (20%)
**PTA site**	
ATK	58 (63%)
BTK	87 (95%)

**Table 3:** Cox proportional hazards regression variables in a univariate model for each of the three endpoints. Results are reported as hazards ratios (HR) and associated 95% confidence intervals (CI).

**Healing Within 3 Months**	**HR**	**95% CI**	***p***
Albumin	1.709	1.181–2.473	0.0045
Lymphocytes	1.396	1.089–1.789	0.0085
Monocytes	1.152	1.023–1.297	0.0196
sICAM-1 ng/mL	0.764	0.604–0.965	0.0239
Endothelin-1 pg/mL	0.208	0.048–0.901	0.0358
**New revascularization**	**HR**	**95% CI**	***p***
PAI-1 ng/mL	1.893	1.127–3.179	0.0159
Endothelin-1 pg/mL	7.457	1.093–50.859	0.0402
Atrial fibrillation	2.376	1.028–5.495	0.043
**New lesions or recurrences**	**HR**	**95% CI**	***p***
Monocytes	1.268	1.12–1.435	0.001
IL-10 pg/mL	42.136	3.799–467.359	0.002
Thrombomodulin ng/mL	0.453	0.258–0.797	0.006
Atrial fibrillation	2.429	1.22–4.834	0.012
sCD40L pg/mL	1.744	1.055–2.882	0.030
IL-1RA pg/mL	1.169	1.006–1.359	0.042

## Data Availability

Data from this study will be made available upon reasonable request.

## Supplementary Materials

The supporting information can be downloaded at: https://www.mdpi.com/article/10.3390/ijms231810641/s1.
