# Supplementary material for: Circulating Biomarkers of Endothelial Dysfunction and Inflammation in Predicting Clinical Outcomes in Diabetic Patients with Critical Limb Ischemia
Source: Int J Mol Sci. 2022 Sep 13;23(18):10641. doi: 10.3390/ijms231810641 (PMC9506462; doi:10.3390/ijms231810641)
Supplement: Supplementary file 1 [file ijms-23-10641-s001.zip › ijms-1896956-supplementary.pdf]

International Journal of
Molecular Sciences

MDPI

Supplemental Materials

Clustering and partition tree

The training set contained 65 cases, the clusters had the following proportions: cluster 1 = 23.08%, cluster 2 = 64.62% and cluster 3 = 12.31%. The test set contains 27 cases, the clusters have the following proportions: cluster 1 = 22.22%, cluster 2 = 66.67% and cluster 3 = 11.11%. The maximum accuracy value was used to select the optimal model. The obtained complexity parameter cp = 0.244 was used for the construction of the decision tree. The number of splits is equal to two. The performance characteristics of the decision tree were calculated on the confusion matrix. The overall accuracy of the model is 0.812 (95% c.i. = 0.6192,0.937). The sensitivity measures per class are equal to 0.667, 0.833, and 1 for classes 1,2, and 3 respectively. The specificity measures for the classes are equal to 0.952, 0.778, and 0.917 for classes 1,2, and 3 respectively. The positive predictive value for the classes is equal to 0.8, 0.882, and 0.6 for classes 1,2, and 3 respectively. The negative predictive value for the classes is 0.909, 0.7 and 0.1 for classes 1,2 and 3 respectively.

Table S1. Descriptive statistics clusters outcome 1 (Wound healing within 3 month).

	Cluster 1 (n =51)	Cluster 2 (n =41)	Overall (n = 92)	p
Age	73.00 [68.00- 80.50]	72.00 [68.00- 77.00]	72.50 [68.00- 78.00]	0.335
Sex Male	40 (78.4%)	31 (75.6%)	71 (77.2%)	0.806
BMI	26.30 [24.44- 28.95]	26.89 [25.47- 29.30]	26.68 [24.93- 29.30]	0.460
Smoking status				0.815
Never	14 (27.5%)	13 (32.5%)	27 (29.7%)	
Past	31 (60.8%)	24 (60.0%)	55 (60.4%)	
Active	6 (11.8%)	3 (7.5%)	9 (9.9%)	
Diabetes type II	48 (96.0%)	37 (90.2%)	85 (93.4%)	0.193
Insulin therapy	38 (79.2%)	29 (72.5%)	67 (76.1%)	0.616
Carotid artery disease	5 (9.8%)	3 (7.3%)	8 (8.7%)	0.728
Stroke or TIA	4 (8.0%)	4 (9.8%)	8 (8.8%)	1.000
Heart failure	7 (13.7%)	1 (2.4%)	8 (8.7%)	0.071
Coronary artery disease	22 (43.1%)	20 (48.8%)	42 (45.7%)	0.675
Previous myocardial_infarction	4 (7.8%)	6 (14.6%)	10 (10.9%)	0.332
Valvular prosthesis	1 (2.0%)	3 (7.3%)	4 (4.4%)	0.327
Atrial fibrillation	17 (33.3%)	5 (12.2%)	22 (23.9%)	0.026
**Biomarkers**				
sCD40L pg/mL	14.10 [9.05- 19.73]	10.78 [6.42- 15.24]	11.73 [7.32- 17.91]	0.070
IFN-α2 pg/mL	8.00 [8.00- 8.00]	8.00 [8.00- 8.00]	8.00 [8.00- 8.00]	0.621
IFN-γ pg/mL	1.28 [1.21- 1.28]	1.28 [1.28- 9.48]	1.28 [1.28- 3.00]	0.013
IL-1Ra pg/mL	7.58 [3.60- 13.46]	7.38 [4.26- 10.48]	7.56 [3.82- 12.47]	0.833
IL-2 pg/mL	0.64 [0.64- 0.64]	0.64 [0.64- 0.64]	0.64 [0.64- 0.64]	0.900
IL-4 pg/mL	1.48 [0.64- 3.57]	1.60 [0.64- 4.00]	1.60 [0.64- 3.86]	0.875
IL-5 pg/mL	3.70 [2.30- 7.09]	3.18 [2.09- 6.54]	3.54 [2.29- 6.88]	0.369
IL-6 pg/mL	5.33 [2.37- 8.51]	4.67 [1.98- 9.50]	4.83 [2.15- 9.08]	0.687
IL-10 pg/mL	2.56 [2.56- 2.56]	2.56 [2.56- 2.56]	2.56 [2.56- 2.56]	0.534
IL-13 pg/mL	6.40 [6.40- 6.40]	6.40 [6.40- 10.55]	6.40 [6.40- 10.12]	0.348
IL-18 pg/mL	34.25 [26.02- 46.81]	30.23 [17.96- 45.69]	34.01 [23.73- 46.45]	0.384
TNF-α ng/mL	18.24 [14.34- 29.58]	20.14 [10.10- 40.66]	19.14 [12.00- 30.75]	0.942
Angiopoietin-2 ng/mL	2.98 [2.35- 4.52]	2.59 [1.65- 4.16]	2.92 [1.92- 4.51]	0.260
Endoglin ng/mL	1.61 [1.34- 2.17]	1.74 [1.25- 2.13]	1.71 [1.31- 2.15]	0.942

Endothelin-1 pg/mL.	2.70 [2.70- 2.70]	2.70 [1.92- 2.70]	2.70 [2.70- 2.70]	0.188
E-Selectin ng/mL	32.97 [24.20- 41.04]	34.19 [23.37- 42.37]	34.19 [23.82- 42.13]	0.847
Thrombomodulin ng/mL	1.10 [0.75- 1.54]	1.25 [0.85- 1.99]	1.13 [0.82- 1.82]	0.184
sRAGE pg/mL.	62.17 [30.21- 97.14]	49.51 [35.85- 92.81]	56.21 [31.90- 97.12]	0.707
sICAM.1 ng/mL	189.40 [140.83- 252.43]	204.75 [179.60- 289.79]	201.71 [154.75- 263.22]	0.237
P-Selectin ng/mL	119.01 [83.66- 139.79]	92.43 [63.86- 127.10]	102.77 [74.50- 139.38]	0.206
sVCAM.1 ng/mL	1049.39 [874.59- 1269.58]	1028.05 [876.21- 1199.38]	1036.44 [870.09- 1239.68]	0.621
PAI-1 ng/mL	186.80 [158.46- 262.14]	195.70 [138.07- 235.45]	189.48 [147.70- 241.46]	0.367
vWF µg/mL.	26.43 [19.41- 38.28]	28.95 [17.03- 41.14]	26.85 [17.90- 40.02]	0.858
**Laboratory**				
White blood cells	9.10 [7.65- 10.35]	8.80 [6.90- 11.00]	9.05 [7.25- 10.72]	0.643
Haemoglobin	12.40 [11.60- 13.60]	11.20 [10.40- 12.60]	12.10 [10.80- 13.22]	0.010
Platelets	269.00 [204.50- 331.00]	295.00 [240.00- 331.00]	277.50 [216.75- 333.00]	0.229
Neutrophils	6.40 [5.20- 7.55]	6.60 [4.10- 7.80]	6.40 [4.68- 7.80]	0.795
Limphocytes	1.80 [1.45- 2.15]	1.90 [1.30- 2.20]	1.80 [1.40- 2.20]	0.841
Monocytes	0.70 [0.50- 0.85]	0.60 [0.50- 0.80]	0.70 [0.50- 0.80]	0.361
EGFR (Cock)	55.74 [42.66- 78.81]	63.32 [43.43- 86.24]	58.69 [42.70- 84.93]	0.502
Triglycerides	121.00 [88.50- 175.00]	121.00 [92.00- 150.00]	121.00 [89.75- 161.50]	0.646
HDL	39.00 [33.00- 47.50]	37.00 [34.00- 43.00]	38.00 [33.00- 46.00]	0.385
LDL	65.80 [51.60- 85.40]	65.20 [51.20- 90.20]	65.60 [51.50- 87.80]	0.689
Hba1c	7.27 [6.36- 8.05]	7.37 [6.36- 8.19]	7.32 [6.34- 8.12]	0.759
CRP	0.90 [0.20- 3.20]	1.60 [0.30- 2.80]	1.20 [0.25- 3.20]	0.350
Albumin	3.63 [3.27- 3.95]	3.59 [3.30- 4.06]	3.62 [3.29- 3.98]	0.912

Table S2. Descriptive statistics clusters outcome 2 (New re-vascularization).

	**Cluster 1 (n = 70)**	**Cluster 2 (n = 22)**	**Overall (n =72)**	**p**
Age	71.00 [67.25- 77.75]	75.50 [69.50- 80.50]	72.50 [68.00- 78.00]	0.132
Sex Male	51 (72.9%)	20 (90.9%)	71 (77.2%)	0.090
BMI	26.09 [24.22- 28.93]	27.43 [26.16- 30.15]	26.68 [24.93- 29.30]	**0.033**
Smoking status:				0.402
Never	23 (33.3%)	4 (18.2%)	27 (29.7%)	
Past	39 (56.5%)	16 (72.7%)	55 (60.4%)	
Active	7 (10.1%)	2 (9.1%)	9 (9.9%)	
Diabetes type II	65 (92.9%)	20 (95.2%)	85 (93.4%)	0.803
Insulin therapy	51 (75.0%)	16 (80.0%)	67 (76.1%)	0.771
Carotid artery disease	4 (5.7%)	4 (18.2%)	8 (8.7%)	0.090
Stroke or TIA	6 (8.7%)	2 (9.1%)	8 (8.8%)	1.000
Heart failure	4 (5.7%)	4 (18.2%)	8 (8.7%)	0.090
Coronary artery disease	28 (40.0%)	14 (63.6%)	42 (45.7%)	0.085
Myocardial infarction	6 (8.6%)	4 (18.2%)	10 (10.9%)	0.243
Valvular prosthesis	3 (4.3%)	1 (4.8%)	4 (4.4%)	1.000
Atrial fibrillation	0 (0.0%)	22 (100.0%)	22 (23.9%)	**<0.001**
**Biomarkers**				
sCD40L pg/mL	11.98 [7.72- 17.48]	11.28 [5.04- 18.52]	11.73 [7.32- 17.91]	0.713
IFN-α2 pg/mL	8.00 [8.00- 8.00]	8.00 [8.00- 8.00]	8.00 [8.00- 8.00]	0.454
IFN-γ pg/mL	1.28 [1.28- 3.00]	1.28 [1.01- 2.06]	1.28 [1.28- 3.00]	0.432
IL-1Ra pg/mL	6.51 [3.62- 10.17]	9.50 [5.29- 17.72]	7.56 [3.82- 12.47]	**0.051**
IL-2 pg/mL	0.64 [0.64- 0.64]	0.64 [0.64- 0.64]	0.64 [0.64- 0.64]	**0.042**
IL-4 pg/mL	1.44 [0.64- 3.76]	1.84 [0.88- 5.72]	1.60 [0.64- 3.86]	0.296

IL-5 pg/mL	3.54 [2.15- 6.73]	4.00 [2.59- 7.21]	3.54 [2.29- 6.88]	0.523
IL-6 pg/mL	4.23 [1.90- 7.26]	8.17 [2.87- 21.89]	4.83 [2.15- 9.08]	**0.008**
IL-10 pg/mL	2.56 [2.56- 2.56]	2.56 [2.56- 2.56]	2.56 [2.56- 2.56]	0.937
IL-13 pg/mL	6.40 [6.40- 11.70]	6.40 [6.13- 6.40]	6.40 [6.40- 10.12]	**0.044**
IL-18 pg/mL	29.80 [20.26- 46.45]	36.79 [29.81- 46.69]	34.01 [23.73- 46.45]	0.394
TNF-α ng/mL	17.47 [10.35- 29.90]	23.47 [17.52- 31.83]	19.14 [12.00- 30.75]	0.073
Angiopoietin-2 ng/mL	2.45 [1.71- 3.49]	4.89 [3.47- 9.02]	2.92 [1.92- 4.51]	**<0.001**
Endoglin ng/mL	1.71 [1.28- 2.08]	1.72 [1.51- 2.62]	1.71 [1.31- 2.15]	0.314
Endothelin-1 pg/mL.	2.70 [2.70- 2.70]	2.70 [2.70- 2.70]	2.70 [2.70- 2.70]	0.113
E-Selectin ng/mL	34.36 [23.87- 43.02]	29.38 [23.78- 40.07]	34.19 [23.82- 42.13]	0.418
Thrombomodulin ng/mL	1.19 [0.83- 1.82]	1.05 [0.65- 1.79]	1.13 [0.82- 1.82]	0.486
sRAGE pg/mL.	56.21 [30.78- 92.81]	57.63 [42.86- 137.01]	56.21 [31.90- 97.12]	0.166
sICAM.1 ng/mL	205.41 [159.88- 263.22]	186.92 [139.03- 278.49]	201.71 [154.75- 263.22]	0.558
P-Selectin ng/mL	103.57 [77.70- 136.95]	99.85 [69.29- 158.47]	102.77 [74.50- 139.38]	0.907
sVCAM.1 ng/mL	1036.44 [870.09- 1224.16]	1072.30 [904.39- 1282.74]	1036.44 [870.09- 1239.68]	0.662
PAI-1 ng/mL	178.88 [145.59- 240.35]	223.53 [173.39- 290.62]	189.48 [147.70- 241.46]	0.090
vWF μg/mL.	26.85 [17.70- 38.03]	32.08 [19.88- 47.11]	26.85 [17.90- 40.02]	0.341
**Laboratory**				
White blood cells	9.05 [7.15- 10.80]	9.10 [7.88- 10.60]	9.05 [7.25- 10.72]	0.667
Haemoglobin	12.05 [10.72- 13.12]	12.45 [11.60- 13.52]	12.10 [10.80- 13.22]	0.278
Platelets	276.50 [205.75- 337.00]	282.00 [232.25- 312.75]	277.50 [216.75- 333.00]	0.670
Neutrophils	6.40 [4.28- 7.47]	6.40 [5.48- 8.05]	6.40 [4.68- 7.80]	0.278
Limphocytes	1.90 [1.50- 2.27]	1.65 [1.33- 1.90]	1.80 [1.40- 2.20]	**0.037**
Monocytes	0.65 [0.50- 0.90]	0.70 [0.60- 0.80]	0.70 [0.50- 0.80]	0.552
EGFR (Cock)	55.17 [42.45- 85.81]	66.69 [49.62- 80.42]	58.69 [42.70- 84.93]	0.608
Triglycerides	120.00 [90.50- 154.50]	145.00 [90.25- 166.00]	121.00 [89.75- 161.50]	0.498
HDL	39.00 [34.25- 47.00]	37.00 [30.75- 39.00]	38.00 [33.00- 46.00]	**0.052**
LDL	65.30 [52.50- 89.40]	67.00 [46.55- 82.15]	65.60 [51.50- 87.80]	0.840
Hba1c	7.27 [6.38- 8.08]	7.50 [6.20- 8.71]	7.32 [6.34- 8.12]	0.576
CRP	1.20 [0.20- 3.20]	1.20 [0.40- 3.98]	1.20 [0.25- 3.20]	0.447
Albumin	3.66 [3.33- 4.01]	3.45 [3.08- 3.87]	3.62 [3.29- 3.98]	0.210

Table S3. Descriptive statistics clusters outcome 3 (New lesion or recurrences).

	**Cluster 1 (n = 21)**	**Cluster 2 (n = 60)**	**Cluster 3 (n =11)**	**Overall**	**p**
Age	69.00 [62.00- 71.00]	74.00 [69.00- 79.25]	72.00 [69.00- 76.00]	72.50 [68.00- 78.00]	0.031
Sex Male	18 (85.7%)	44 (73.3%)	9 (81.8%)	71 (77.2%)	0.571
BMI	27.46 [26.12- 31.38]	26.74 [24.69- 28.99]	26.02 [24.04- 26.38]	26.68 [24.93- 29.30]	0.082
Smoking status:					0.199
Never	4 (19.0%)	19 (32.2%)	4 (36.4%)	27 (29.7%)	
Past	12 (57.1%)	36 (61.0%)	7 (63.6%)	55 (60.4%)	
Active	5 (23.8%)	4 (6.8%)	0 (0.0%)	9 (9.9%)	
Diabetes type II	21 (100.0%)	55 (91.7%)	9 (90.0%)	85 (93.4%)	0.568
Insulin therapy	18 (85.7%)	42 (73.7%)	7 (70.0%)	67 (76.1%)	0.523
Carotid artery disease	1 (4.8%)	7 (11.7%)	0 (0.0%)	8 (8.7%)	0.627
Stroke or TIA	1 (5.0%)	5 (8.3%)	2 (18.2%)	8 (8.8%)	0.388
Heart failure	4 (19.0%)	3 (5.0%)	1 (9.1%)	8 (8.7%)	0.096
Coronary artery disease	10 (47.6%)	26 (43.3%)	6 (54.5%)	42 (45.7%)	0.820

Previous myocardial_in-farction	1 (4.8%)	5 (8.3%)	4 (36.4%)	1 (10.9%)	0.030
Valvular prosthesis	0 (0.0%)	2 (3.4%)	2 (18.2%)	4 (4.4%)	0.105
Atrial fibrillation	6 (28.6%)	13 (21.7%)	3 (27.3%)	22 (23.9%)	0.706
**Biomarkers**					
sCD40L pg/mL	23.29 [19.59- 26.07]	10.41 [7.35- 13.61]	4.05 [2.90- 8.79]	11.73 [7.32- 17.91]	<0.001
IFN-α2 pg/mL	8.00 [8.00- 8.00]	8.00 [8.00- 8.00]	8.00 [8.00- 8.00]	8.00 [8.00- 8.00]	0.908
IFN-γ pg/mL	1.28 [1.23- 1.33]	1.28 [1.28- 1.48]	4.90 [1.28- 9.35]	1.28 [1.28- 3.00]	0.198
IL-1Ra pg/mL	8.75 [6.60- 21.71]	6.51 [3.24- 9.87]	6.14 [5.52- 11.68]	7.56 [3.82- 12.47]	0.053
IL-2 pg/mL	0.64 [0.64- 0.64]	0.64 [0.64- 0.64]	0.64 [0.64- 0.64]	0.64 [0.64- 0.64]	0.857
IL-4 pg/mL	4.53 [1.27- 6.53]	1.07 [0.64- 3.10]	2.55 [0.92- 3.36]	1.60 [0.64- 3.86]	0.010
IL-5 pg/mL	3.26 [2.24- 4.58]	3.62 [2.29- 6.93]	5.83 [2.81- 18.88]	3.54 [2.29- 6.88]	0.219
IL-6 pg/mL	9.80 [3.73- 18.31]	4.13 [1.76- 6.87]	4.32 [2.80- 6.95]	4.83 [2.15- 9.08]	0.053
IL-10 pg/mL	2.56 [2.56- 30.49]	2.56 [2.56- 2.56]	2.56 [2.56- 2.56]	2.56 [2.56- 2.56]	0.113
IL-13 pg/mL	6.40 [5.95- 12.92]	6.40 [6.40- 6.40]	6.40 [5.77- 9.30]	6.40 [6.40- 10.12]	0.973
IL-18 pg/mL	30.69 [22.76- 48.32]	34.01 [23.84- 45.69]	34.54 [29.97- 37.62]	34.01 [23.73- 46.45]	0.882
TNF-α ng/mL	24.88 [18.52- 38.99]	16.67 [10.10- 26.79]	25.21 [13.44- 45.58]	19.14 [12.00- 30.75]	0.022
Angiopoietin-2 ng/mL	3.26 [2.55- 5.42]	2.88 [1.90- 3.70]	3.09 [1.36- 6.59]	2.92 [1.92- 4.51]	0.301
Endoglin ng/mL	1.61 [1.33- 2.07]	1.61 [1.27- 1.95]	2.49 [2.25- 3.08]	1.71 [1.31- 2.15]	0.003
Endothelin-1 pg/mL.	2.70 [2.70- 2.70]	2.70 [2.70- 2.70]	2.70 [2.12- 2.70]	2.70 [2.70- 2.70]	0.540
E-Selectin ng/mL	35.23 [28.14- 45.50]	34.19 [23.37- 42.37]	28.00 [21.82- 34.83]	34.19 [23.82- 42.13]	0.291
Thrombomodulin ng/mL	0.64 [0.48- 1.02]	1.24 [0.90- 1.57]	2.97 [2.64- 3.36]	1.13 [0.82- 1.82]	<0.001
sRAGE pg/mL.	37.40 [25.20- 56.28]	72.42 [38.62- 113.48]	52.86 [40.28- 132.01]	56.21 [31.90- 97.12]	0.041
sICAM.1 ng/mL	223.36 [126.90- 353.66]	196.50 [158.03- 247.10]	239.26 [204.74- 303.94]	201.71 [154.75- 263.22]	0.384
P-Selectin ng/mL	131.17 [114.16- 173.14]	91.41 [69.89- 124.93]	60.13 [45.17- 124.35]	102.77 [74.50- 139.38]	0.001
sVCAM.1 ng/mL	931.54 [757.10- 1059.31]	1052.86 [914.94- 1239.57]	1401.06 [1217.33- 1502.07]	1036.44 [870.09- 1239.68]	0.001
PAI-1 ng/mL	230.38 [211.09- 281.84]	182.59 [139.01- 239.26]	153.60 [119.21- 173.93]	189.48 [147.70- 241.46]	0.002
vWF µg/mL.	31.05 [25.04- 38.65]	23.52 [16.90- 40.84]	36.05 [27.48- 43.03]	26.85 [17.90- 40.02]	0.177
**Laboratory**					
White blood cells	10.90 [9.10- 12.50]	8.85 [6.97- 10.22]	8.50 [6.65- 9.40]	9.05 [7.25- 10.72]	0.001
Haemoglobin	12.20 [10.60- 14.30]	12.15 [10.88- 13.12]	11.20 [10.70- 12.30]	12.10 [10.80- 13.22]	0.391
Platelets	354.00 [269.00- 427.00]	274.50 [214.00- 314.50]	213.00 [179.00- 267.00]	277.50 [216.75- 333.00]	0.001
Neutrophils	7.80 [6.40- 8.60]	5.80 [4.10- 7.32]	6.00 [4.65- 7.00]	6.40 [4.68- 7.80]	0.002
Limphocytes	1.80 [1.50- 2.00]	1.90 [1.48- 2.30]	1.40 [1.30- 1.75]	1.80 [1.40- 2.20]	0.130
Monocytes	0.90 [0.70- 1.00]	0.60 [0.50- 0.80]	0.60 [0.40- 0.65]	0.70 [0.50- 0.80]	0.001
EGFR (Cock)	74.86 [48.84- 94.70]	53.24 [40.12- 79.70]	55.74 [40.98- 70.25]	58.69 [42.70- 84.93]	0.059
Triglycerides	121.00 [93.00- 218.00]	127.00 [92.75- 162.50]	107.00 [67.50- 122.50]	121.00 [89.75- 161.50]	0.135
HDL	38.00 [29.00- 44.00]	38.50 [34.75- 47.00]	37.00 [34.00- 38.00]	38.00 [33.00- 46.00]	0.520
LDL	59.20 [48.20- 98.00]	69.00 [52.70- 84.60]	63.40 [42.50- 71.60]	65.60 [51.50- 87.80]	0.552
Hba1c	7.55 [6.91- 8.37]	7.50 [6.66- 8.12]	6.27 [5.54- 6.54]	7.32 [6.34- 8.12]	0.015
CRP	2.10 [0.73- 5.25]	0.90 [0.20- 2.73]	1.30 [0.25- 1.90]	1.20 [0.25- 3.20]	0.124

Albumin	3.48 [3.04- 3.93]	3.68 [3.36- 4.00]	3.62 [3.15- 3.90]	3.62 [3.29- 3.98]	0.241
